# Normative Data of the Trail Making Test Among Urban Community-Dwelling Older Adults in Japan

**DOI:** 10.3389/fnagi.2022.832158

**Published:** 2022-05-25

**Authors:** Hiroyuki Suzuki, Naoko Sakuma, Momoko Kobayashi, Susumu Ogawa, Hiroki Inagaki, Ayako Edahiro, Chiaki Ura, Mika Sugiyama, Fumiko Miyamae, Yutaka Watanabe, Shoji Shinkai, Shuichi Awata

**Affiliations:** ^1^Research Team for Social Participation and Community Health, Tokyo Metropolitan Institute of Gerontology, Tokyo, Japan; ^2^Research Team for Promoting Independence and Mental Health, Tokyo Metropolitan Institute of Gerontology, Tokyo, Japan; ^3^Gerodontology, Department of Oral Health Science, Faculty of Dental Medicine, Hokkaido University, Sapporo, Japan; ^4^Faculty of Nutrition Sicences, Kagawa Nutrition University, Sakado, Japan; ^5^Social and Human Sciences, Tokyo Metropolitan Institute of Gerontology, Tokyo, Japan; ^6^Integrated Research Initiative for Living Well with Dementia, Tokyo Metropolitan Institute of Gerontology, Tokyo, Japan

**Keywords:** Trail Making Test, normative data, older adults, cognitive decline, neurocognitive assessment

## Abstract

**Introduction:**

Population aging is likely to increase the number of people with dementia living in urban areas. The Trail Making Test (TMT) is widely used as a cognitive task to measure attention and executive function among older adults. Normative data from a sample of community-dwelling older adults are required to evaluate the executive function of this population. The purpose of this study was to examine the Trail Making Test completion rate and completion time among urban community-dwelling older adults in Japan.

**Methods:**

A survey was conducted at a local venue or during a home visit (*n* = 1,966). Cognitive tests were conducted as a part of the survey, and TMT Parts A (TMT-A) and B (TMT-B) were completed after the completion of the Japanese version of the Mini-Mental State Examination (MMSE-J). Testers recorded TMT completion status, completion time, and the number of errors observed.

**Results:**

In the TMT-A, 1,913 (99.5%) participants understood the instructions, and 1,904 (99.1%) participants completed the task within the time limit of 240 s. In the TMT-B, 1,839 (95.9%) participants understood the instructions, and 1,584 (82.6%) participants completed the task within the time limit of 300 s. The completion rate of TMT-B was 90.2 and 41.8% for participants with an MMSE-J score of >23 points and ≦23 points, respectively. Results of multiple regression analyses showed that age, education, and the MMSE-J score were associated with completion time in both TMTs.

**Conclusion:**

In both TMTs, completion time was associated with age, education, and general cognitive function. However, not all participants completed the TMT-B, and the completion rate was relatively low among participants with low MMSE-J scores. These findings may help interpret future TMT assessments.

## Introduction

Population aging is a global phenomenon, occurring at high rates in Eastern and South-Eastern Asia, Latin America, and the Caribbean (United Nations, The Population Division of the Department of Economic and Social Affairs, 2020). Japan is one of the most rapidly aging societies in the world; even in urban areas where the current aging rate is relatively low, the size of the aging population is expected to rapidly increase in the near future (Cabinet Office Japan, 2018). Approximately 30% of Japan’s total population lives around Tokyo (Statistics Bureau of Japan, 2021). Population aging is likely to increase the number of people with dementia living in urban areas (Asada, 2013). This study on the prevalence of dementia in Japan estimates that the prevalence increases with the inclusion of urban areas, and that the overall prevalence in Japan is about 15%. Progress in dementia prevention and management requires insight into cognitive function in older adults; in particular, the faculties that directly affect the activities of daily living. Specifically, deficits in the attention and executive function necessary to smoothly process things according to the procedures presented can impair quality of life in urban areas where the environment is prone to change.

The Trail Making Test (TMT) is widely used as a cognitive task to measure attention and executive function among older adults (Arbuthnott and Frank, 2000; Tombaugh, 2004). It involves connecting randomly arranged circles with a pencil, and comes in Parts A (TMT-A) and B (TMT-B), which are used for functional evaluation of patients with brain injury (Reitan, 1958). In TMT-A, numbers are written in circles, and test takers are asked to connect the numbers in ascending order. In TMT-B, numbers or letters are written in circles, and test takers are asked to connect them alternately and in ascending order. In both TMTs, the time to completion is the main evaluation index. Processing speed such as that required for visual search is strongly reflected in the results of TMT-A, and working memory and cognitive flexibility are involved in TMT-B (Lezak et al., 2012). The TMT is not only sensitive to changes in cognitive function due to brain injury (Reitan, 1958); it may also detect those that occur due to aging and education (Hashimoto et al., 2006; Specka et al., 2021). The TMT is widely used in the evaluation of cognitive function in older adults, including evaluation indicators in intervention studies (Jacobs et al., 2020; Suzuki et al., 2020), driving performance in older adults (Vaucher et al., 2014), and studies examining the relationship between physiological indicators and attention function (Uchida et al., 2020). Both TMT scores are sensitive to progressive cognitive decline associated with dementia (Greenlief et al., 1985). Older adults with poor TMT-B performance have problems performing the activities of daily living (Bell-McGinty et al., 2002).

Normative data from a sample of community-dwelling older adults are required to evaluate the executive function of this population. Some studies have examined the normative values of the TMT completion time in healthy older adults (Tombaugh, 2004; Mitrushina et al., 2005; Cangoz et al., 2009; Fallman et al., 2020), but few reports have accounted for the TMT completion status (Seo et al., 2006; Wei et al., 2018; Specka et al., 2021). TMT-B involves complex processes and understanding what percentage of test takers complete the test is necessary to contextualize completion time values in community-dwelling older adults. Excluding data from adults unable to complete TMT-B may result in a standardized TMT-B value that overestimates population ability to complete this test. By classifying the completion status and examining the characteristics of older adults who have difficulty understanding the instructions and completing it within the time limit, it will be possible to examine appropriate methods of evaluating executive function using the TMT-B and reducing the burden. In addition, although the TMT completion time is affected by variables such as age, education, and general cognitive function, few previous studies have reported on the impact of these characteristics in the context of large-scale normative data for community-dwelling older adults in urban areas.

This study aimed to examine the normative TMT completion rate and completion time values for older adults living in urban areas using data from the Takashimadaira study (Iwasaki et al., 2020), which is a large-scale survey of community-dwelling older adults. Normative data from large-scale surveys may contribute to executive function evaluations using the TMT in older adults living in urban areas. Simultaneously evaluating the TMT completion status, age, sex, education, and general cognitive function of participants allows to examine any associations between participant characteristics and test performance. This study is the first to comprehensively examine the association between TMT and these variables using large-scale data in older adults. By using large-scale data to present normative values based on each attribute associated with TMT performance, it is possible to detect whether or not attention and executive function are lower than age-appropriate for a diverse group of older adults in the community. This contributes to early screening for cognitive decline in old age, such as mild cognitive impairment.

## Materials and Methods

This study was conducted from 2016 to 2017 in Takashimadaira area of Itabashi Ward, which is located on the north side of Tokyo, Japan. Takashimadaira is a large housing complex that was built during the 70 s, which was a high-growth period in Japan. Within Itabashi-ku, Takashimadaira is home to a high percentage of adults aged ≥65 years and the aging of the urban population is occurring ahead of other areas (total population is approximately 32,500). A mail survey was conducted as a primary survey of all adults aged ≥70 years (*n* = 7,614) living in this area. The people who responded to the first mail survey (*n* = 5,432) were invited by letter to participate in the second survey, which involved face-to-face health-related interviews. The second survey was conducted at a local venue or during a home visit. A total of 1,966 people who responded to the TMT were eligible for this study. The participants’ demographic characteristics are presented in Table 1. Because we wanted to examine completion status and times for a diverse population with mixed characteristics in the community, we did not have any exclusion criteria in our sample. For normative data, not only data from all participants but also data excluding participants with neurological symptoms were created.

**TABLE 1:** Characteristics of the study population.

			Age group				
		Total	70–74	75–79	80–84	85–90	90–100
Participants	*n* (%)	1,966 (100)	600 (30.5)	654 (33.3)	465 (23.7)	182 (9.3)	65 (3.3)
Age	Mean (SD)	78.0 (5.4)	72.3 (1.4)	76.9 (1.4)	81.7 (1.4)	86.6 (1.3)	91.9 (2.3)
Gender	Female, %	59.9	56.7	62.4	58.3	62.1	69.2
Education (years)	Mean (SD)	12.3 (2.8)	12.5 (2.4)	12.4 (2.5)	12.4 (3.2)	11.8 (3.4)	10.7 (3.3)
12 years or less	%	68.5	67.3	69.9	65.3	72.9	75.4
MMSE-J (scores)	Mean (SD)	26.3 (3.4)	27.4 (2.4)	26.6 (3.2)	25.8 (3.4)	24.9 (3.8)	21.9 (5.8)
Score 23 or less	%	16.3	7.9	13.7	21.2	27.5	50.8
Survey location (venue or home)	Venue, %	66.0	74.5	68.5	62.2	50.0	35.4
Neurological symptoms	*n*	255	48	83	72	41	11
History of stroke	*n*	178	34	59	55	25	5
Parkinson’s disease	*n*	18	4	7	3	4	0
Dementia	*n*	42	6	9	10	11	6
Current depression	*n*	29	5	10	6	5	3

*MMSE-J, Japanese version of the mini-mental state examination.*

### Data Collection and Variables

Data on demographic characteristics were collected using the first mail survey. Cognitive tests were conducted as part of the secondary survey, and TMTs were performed after the Mini-Mental State Examination (MMSE-J) (Folstein et al., 1975; Sugishita and Hemmi, 2010). The cognitive tests were conducted by trained nurses or psychologists. There were 69 missing on information of education and 16 missing on MMSE-J due to response refusals and survey errors. In one case, both education and MMSE-J information was missing.

The Japanese version of the TMT used in this study was based on the original version (Reitan, 1958) and had been used in previous studies (Suzuki et al., 2014, 2020). Both parts of the TMT consisted of 25 scattered circles drawn on the examination paper. In TMT-A, the circles were numbered from 1 to 25, and the participants were asked to draw lines to connect the presented numbers in ascending order as quickly as possible. In TMT-B, the circles included either numbers from 1 to 13 or the first 12 characters of the Japanese Hiragana alphabet. Participants were required to alternately connect numbers and characters. Practice tests were performed for both parts and involved eight circles with a layout different from that used in the final test; all participants received instructions on how to perform the task until they understood it; participants that failed to understand the training task did not perform the final tests and their outcome was recorded separately. During the final test, the time required to complete the task was recorded (seconds), as were participant errors; when errors were noted, participants were asked to stop the task and then resume it from the place where they had made the error. The time limit was set to 240 and 300 s for Parts A and B, respectively. The implementation status, completion time, and number of errors were recorded by the tester.

We distinguished between two types of reasons for failure to complete the task to understand the type of difficulties associated with it: failure to understand the instructions and failure to complete the task within the time limit despite understanding the instructions. These items were recorded by the testers and cross-checked by other testers. The time required by participants to complete the test was aggregated to obtain a normative value.

### Statistical Analysis

The participants’ characteristics and normative test values were examined. Density plots are shown for all participants and those stratified to exclude older adults with neurological symptoms (history of stroke, Parkinson’s disease, dementia, and current depression). Correlation analyses were performed between the demographic variables and completion times on TMT-A and TMT-B. Multiple regression analysis was performed to examine the impact of age, years of education, sex, and MMSE-J scores on TMT completion time; these analyses were performed separately for all participants or the participants without neurological symptoms. Log-transformed completion times were used for multiple regression analysis. Chi-square tests and analyses of variance were performed to compare the characteristics of participants among the completed, not completed within the time limit, and failure to understand the instructions groups. Bonferroni’s method was used for multiple comparisons. All analyses were performed using IBM SPSS Statistics for Windows version 25 (IBM Corp., Tokyo, Japan).

### Ethics Approval and Participants Consent Statement

This study was conducted in accordance with the ethical principles of the Declaration of Helsinki and was approved by the Ethics Committee of the Tokyo Metropolitan Institute of Gerontology (approval number 9 and 31 in 2016). Written informed consent was obtained from all the participants prior to the survey.

## Results

### Completion Rate of the Trail Making Test’s in Older Adults

**FIGURE 1:**
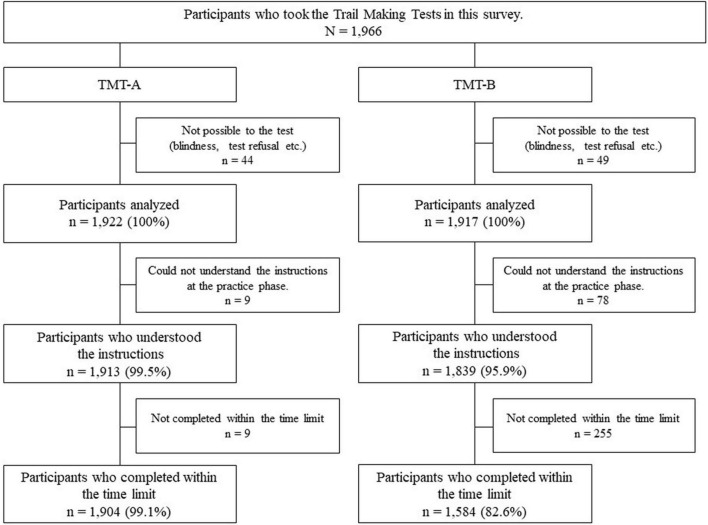
Flowchart on implementation status of the Trail Making Tests.

Data invalid due to tester errors and those from participants that could not perform the TMT due to sensory or physical dysfunction were excluded. A total of 1,922 and 1,917 participants provided valid TMT-A and TMT-B data, respectively (shown in Figure 1). For TMT-A, 1,913 (99.5%) participants understood and undertook the task, and 1,904 (99.1%) participants completed it within the time limit. For TMT-B, 1,839 (95.9%) participants understood the procedure, and 1,584 (82.6%) participants completed it within the time limit. Most participants were able to complete TMT-A, and 17.4% of the participants were not able to complete TMT-B. Participants who completed TMT-B within the time limit were younger than the non-completers and had more years of education and higher MMSE-J scores (*p* < 0.01). Among the non-completers, the participants who could not understand the instructions were older and had a lower MMSE-J score (*p* < 0.01). No sex-based differences were found among the three groups (Table 2). Of the participants who could not understand the instructions, 34.6% had some neurological condition. Among the participants who were unable to complete the task within the time limit, 22.7% were participants with some neurological condition. Participants with a history of stroke were more likely to be able to complete the task within the time limit (*p* < 0.01).

**TABLE 2:** Characteristics of completer and non-completer in Trail Making Test part B.

		(a) Completed within a time limit	(b) Not completed within a time limit	(c) Could not understand the instructions	*P*-value^1^	Multiple comparison^2^
		*n* = 1584	*n* = 255	*n* = 78		
Age	Mean (SD)	77.2 (4.8)	81.0 (5.6)	84.2 (6.7)	<0.01	a <b < c
	Min-max	70–94	70–96	72–100		
Gender	Female, %	59.2%	63.5%	61.5%	n.s.	
Education (years)	Mean (SD)	12.5 (2.7)	11.2 (3.2)	11.4 (3.2)	<0.01	a > b, c
	Min-max	3–29	6–27	6–20		
MMSE-J (scores)	Mean (SD)	27.2 (2.3)	23.7 (3.5)	18.9 (5.3)	<0.01	a > b > c
	Min-max	16–30	12–30	4–28		
Neurological symptoms	*n* (%)	159 (10.0)	58 (22.7)	27 (34.6)	<0.01	a > b > c
History of stroke	*n* (%)	127 (8.0)	34 (13.3)	10 (12.8)	<0.01	a > b > c
Parkinson’s disease	*n* (%)	10 (0.6)	3 (1.2)	4 (5.1)	n.s.	
Dementia	*n* (%)	12 (0.8)	14 (5.5)	13 (16.7)	n.s.	
Current depression	*n* (%)	13 (0.8)	11 (4.3)	5 (6.4)	n.s.	

*MMSE-J, Japanese version of the mini-mental state examination; SD, standard deviation. ^1^The chi-square test was used to test association among categorical variables, and analysis of covariance was used to compare the means of continuous variables. ^2^The Bonferroni’s method was used for multiple comparison. Multiple comparisons were made using Ryan’s nominal levels for categorical variables.*

### Normative Data of the Trail Making Tests in Older Adults

**FIGURE 2:**
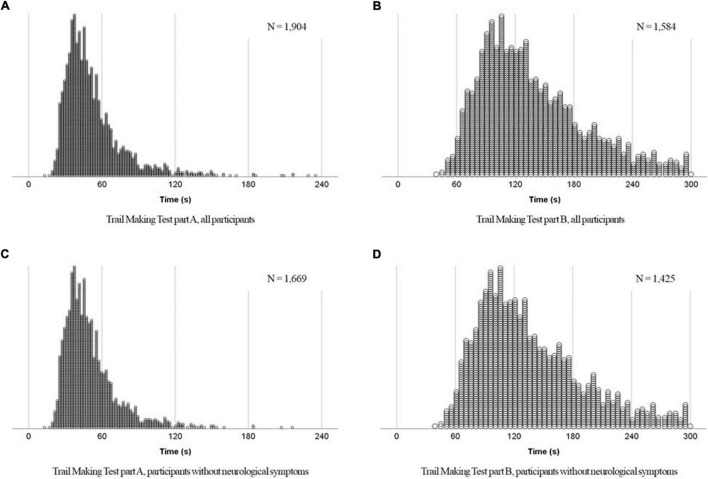
Density plots of completion time of the Trail Making Tests.

Trail Making Test completion time values are shown in Table 3. The density plots are shown in Figure 2. The mode, median, and mean TMT-A completion time values were 37, 46, and 52.7 s, respectively, indicating a log-normal distribution (shown in Figure 2A). TMT-B performance values also showed a log-normal distribution, with the mode, median, and mean values of 107.0, 126.5, and 137.4 s (shown in Figure 2B). The shape of the distribution did not change when the elderly with neurological symptoms were excluded (shown in Figures 2C,D). Details of the normative data are available in Supplementary Material. Data excluding participants with neurological symptoms showed a completion rate of 99.5% for TMT-A and 85.2% for TMT-B (shown in Supplementary Appendix 2A,B).

**TABLE 3:** Summary of the normative data in Trail Making Test part A and B.

			Complete (%)	Completion times (seconds)						With error (%)
		*n*		Mean	SD	Median	Mode	Min	Max	
**TMT-A**										
Total		1,922	99.1	52.7	25.7	46	37	13	235	6.7
Gender	Male	771	99.4	52.6	24.9	46	45	17	216	7.2
	Female	1,151	98.9	52.7	26.2	46	35	13	235	6.4
Education (years)	>12	586	99.5	47.0	21.2	42	37	20	235	5.1
	≦12	1,270	99.0	54.7	26.6	47	35	13	229	7.2
MMSE-J (scores)	>23	1,610	100.0	48.8	21.4	44	35	13	235	6.0
	≦23	296	93.9	75.2	35.3	67	55	29	229	10.8
**TMT-B**										
Total		1,917	82.6	137.4	53.4	126.5	107	39	298	48.2
Gender	Male	769	84.0	140.5	53.0	130	84	49	296	47.8
	Female	1,148	81.7	135.3	53.6	123	124	39	298	48.4
Education (years)	>12	584	89.6	126.5	50.9	115	79	49	297	42.8
	≦12	1,267	81.0	142.8	53.9	131	115	39	298	50.7
MMSE-J (scores)	>23	1,602	90.2	133.1	50.9	122	107	39	298	47.2
	≦23	299	41.8	188.3	56.0	183	152	73	297	60.0

*MMSE-J, Japanese version of the mini-mental state examination; SD, standard deviation.*

### Relationships Between Completion Times and Demographic Characteristics

Multiple regression analyses were used to examine the impact of demographic characteristics on TMT-A and TMT-B completion times (Table 4). For all multiple regression analyses, residual histograms and plots confirmed that there were no problems with normality and homoscedasticity. TMT-A completion time was associated with MMSE-J scores (β = −0.33, *p* < 0.01) more strongly than it was associated with age (β = 0.27, *p* < 0.01) or education (β = −0.13, *p* < 0.01). TMT-B completion time was associated with MMSE-J scores (β = −0.29, *p* < 0.01). Similarly, among cognitively healthy older adults, MMSE-J scores (β = −0.30, *p* < 0.01) was associated with the TMT completion time more strongly than age (β = −0.26, *p* < 0.01). Sex was associated with TMT-B (β = −0.08, *p* < 0.01) but not with TMT-A completion times (β = −0.02, *p* = 0.59). In the overall sample and among cognitively healthy older adults, males performed more slowly than females. There was a moderate correlation between TMTs-A and TMT-B completion time (*r* = 0.44).

**TABLE 4:** Results of the multiple regression analyses for completion time of TMT part A and B.

	Variables	*B*	SE	β	*t*	*p*-value
TMT-A (all participants)	Age (years)	0.01	0.00	0.27	12.97	<0.01
	Gender (male)	–0.01	0.01	–0.02	–1.18	0.24
	Education (years)	–0.01	0.00	–0.13	–6.00	<0.01
	MMSE-J (scores)	–0.02	0.00	–0.33	–15.52	<0.01
TMT-A (participants without neurological symptoms^1^)	Age (years)	0.01	0.00	0.28	12.12	<0.01
	Gender (male)	–0.01	0.01	–0.03	–1.45	0.15
	Education (years)	–0.01	0.00	–0.14	–6.32	<0.01
	MMSE-J (scores)	–0.02	0.00	–0.28	–12.08	<0.01
TMT-B (all participants)	Age (years)	0.01	0.00	0.26	11.46	<0.01
	Gender (male)	–0.03	0.01	–0.09	–3.81	<0.01
	Education (years)	–0.01	0.00	–0.18	–7.78	<0.01
	MMSE-J (scores)	–0.02	0.00	–0.29	–12.53	<0.01
TMT-B (participants without neurological symptoms^1^)	Age (years)	0.01	0.00	0.26	11.03	<0.01
	Gender (male)	–0.03	0.01	–0.08	–3.15	<0.01
	Education (years)	–0.01	0.00	–0.18	–7.33	<0.01
	MMSE-J (scores)	–0.02	0.00	–0.30	–12.15	<0.01

*SE, standard error; MMSE-J, Japanese version of the mini-mental state examination. ^1^Excluded people with a history of stroke, Parkinson’s disease, dementia, and current depression.*

## Discussion and Conclusion

In this study, we examined the normative values of the TMT-A and TMT-B completion time, and their association with participant age, education, sex, and general cognitive function among community-dwelling older adults living in urban areas. While the completion rate of TMT-A was high, approximately 4% of the participants failed to understand TMT-B instructions, and 13% could not complete the task within the time limit. The distribution of the TMT completion time values of the participants who completed it was right-skewed. The mean TMT completion time values were lower than the median values; this finding is consistent with that of previous studies (Tombaugh, 2004). However, these distribution biases are due to the influence of the older adults who are taking time to complete, and even limited to healthy older participants, right skewed distributions were not changed. Data excluding those with neurological symptoms showed higher completion rates for both TMT-A and B, but the difference was small for TMT-A (0.4 points better for TMT-A and 2.6 points better for TMT-B). While most older adults whose MMSE-J scores were lower than the cut-off value failed to complete the TMT-B, the TMT-A shows that it can be performed by most older adults with neurological symptoms. In addition, this study cohort included older adults with the MMSE-J scores of <23 points who did not have dementia (Ura et al., 2020). TMT-A can be completed even by older adults experiencing general cognitive function decline, suggesting it may help evaluate attention function in community surveys over time.

Fewer participants with a history of stroke were unable to understand the instructions in TMT-B. A similar, although not significant, trend was observed for Parkinson’s disease and current depression. In the case of these patients, cognitive decline is associated with disease, and an extended time limit may be useful in properly assessing executive function. On the other hand, in the case of dementia, the number of participants who could not complete the task within the time limit or could not understand the instruction was higher. Even within the time limit, the task should be rounded off if it is unlikely that the patient will be able to complete the task, thereby reducing the burden on the participants.

The TMT completion time was associated with age and general cognitive function test scores; this finding is consistent with that of a previous study (Mitrushina et al., 2005). However, the effects of sex were observed in TMT-B and not in TMT-A performance. Regarding sex effects on the MMSE-J scores, sub-group analysis of participants that completed TMT-B revealed that males scored significantly lower than females (data not shown). This finding indicates that men can complete TMT-B, even if their MMSE-J score is low. Consequently, men that completed TMT-B tended to do so more slowly than women, resulting in an association between sex and test completion time. The MMSE-J score and age were strongly associated with the TMT performance in both part A and B. The associations were also found in an analysis that excluded participants with neurological symptoms. These results suggest that the TMTs may help detect age-related changes in attention and executive function before their clinical manifestation.

Age and the MMSE-J scores of TMT-B non-completers were lower than those of completers. TMT-B is a complex task; performance may be affected by cognitive decline. For this reason, TMT-B results were excluded from a study that reported standardized scores for cognitive tests in older adults (Fallman et al., 2020). Overall, 13.9% of the participants who understood TMT-B instructions could not complete the task within the time limit. In studies involving TMT-B, older adults with cognitive impairment may be naturally excluded. In addition, participants with low MMSE scores and expected difficulty in completing TMT-B may experience psychological distress when asked to complete the task (Brayne et al., 2007; Fowler et al., 2012); therefore, this task is not recommended for use with cognitively impaired individuals. Screening tests for mild cognitive impairment evaluate whether TMT-B can be completed with simple instructions (Nasreddine et al., 2005, 2012). This approach may help evaluate executive function without the risk of causing distress.

Half of the participants who completed TMT-B made errors, resulting in the loss of time. Testers should be highly trained to effectively detect and record such errors; these records may help interpret TMT-B results.

To adequately evaluate the TMT normative data, following six key criterion variables are deemed critical (Mitrushina et al., 2005), (1) fifty cases are considered a desirable sample size, (2) information regarding medical and psychiatric exclusion criteria is important, (3) age group intervals, (4) reporting of education levels, (5) reporting of intellectual levels, (6) reporting of means and standard deviations, and preferably ranges, for total time in seconds for each part of the TMT. In this study, criteria (2) through (6) are met. However, there is a subgroup of older participants with fewer than 50 cases. For example, in the TMT-B for ages 90 and older, there are 17 completers with MMSE-J scores of 24 or higher and only 5 completers with scores of 23 or lower (Supplementary Appendix 1B). As sample size decreases, the influence of outliers also increases, resulting in a reversal: the average completion time for the former group was 183.8 s, while the average completion time for the latter group was 158.8 s, with the group with higher cognitive function having a slower completion time. These data should be considered as reference information rather than normative data.

A comparison of the matchable portions of the completion time of the present study with a similar study conducted in Germany for TMT normative data (Specka et al., 2021) showed that the difference was within a few seconds for both TMT-A and TMT-B. The difference in TMT-B was largest for the 70–74 years old with higher education, with a difference of about 8 s, which was slower in the Japanese data. Specka et al. (2021) excluded older adults with mild cognitive impairment in addition to those with neurological symptoms. The Japanese data do not exclude mild cognitive impairment, and this may be reflected in the difference in the normative data. However, in other areas, the differences were only about 3 s, suggesting that there is essentially little effect of cultural differences on the speed of TMT execution.

This study has some limitations. The older age groups in this study were too small for normative data; data after age 85 should be added. The sample size in the 70 s is rich, but may include undetected early dementia and mild cognitive impairment. Accumulation of data separating healthy older adults from mild cognitive impairment could lead to earlier detection of cognitive decline. Although this study used reliable data to establish normative values for older adults living in urban areas, it did not include older adults living in rural areas. Highly educated and high-functioning older adults may be over-represented in urban areas; thus, the presented findings may not apply to the general older adult population. This is a sampling bias for this study, which conducted initial recruitment by mail in an urban area. Recent studies have shown that urban life is more protective against cognitive decline than rural life (Robbins et al., 2019; Hirst et al., 2021). However, this effect tends to only be observed in the early stages; the subsequent decline tends to be rapid (Xiang et al., 2018). Surveys of the general older adult population may result in completion rate, completion time, and error rate values lower than those presently reported. Older people with low MMSE-J scores need to use more accurate criteria to determine normative TMT data.

This study provided comprehensive TMT normative data stratified by age, education, sex, and general cognitive function for older adults living in an urban area of Japan. In both TMTs, completion time was associated with age, education, and general cognitive function. However, not all participants could complete TMT-B, in particular, among those with low MMSE-J scores. These findings may support the interpretation of past and future study results using TMTs.

## Data Availability Statement

The raw data supporting the conclusions of this article will be made available by the authors, without undue reservation.

## Ethics Statement

The studies involving human participants were reviewed and approved by the Ethics Committee of the Tokyo Metropolitan Institute of Gerontology (approval number 9 and 31 in 2016). The patients/participants provided their written informed consent to participate in this study.

## Author Contributions

HS and NS: conceptualization, methodology, project administration, and writing—review and editing. HI, AE, CU, FM, SO, and MK: data curation. SO, NS, and HS: formal analysis. NS, MK, SO, and HS: investigation. YW, SS, and SA: project administration, resources, and supervision. HS: visualization and writing—original draft. All authors read and approved the published version of the manuscript.

## Conflict of Interest

The authors declare that the research was conducted in the absence of any commercial or financial relationships that could be construed as a potential conflict of interest.

## Publisher’s Note

All claims expressed in this article are solely those of the authors and do not necessarily represent those of their affiliated organizations, or those of the publisher, the editors and the reviewers. Any product that may be evaluated in this article, or claim that may be made by its manufacturer, is not guaranteed or endorsed by the publisher.
