# Supplementary material for: Normative Data of the Trail Making Test Among Urban Community-Dwelling Older Adults in Japan
Source: Front Aging Neurosci. 2022 May 25;14:832158. doi: 10.3389/fnagi.2022.832158 (PMC9175082; doi:10.3389/fnagi.2022.832158)
Supplement: Supplementary file 1 [file Data_Sheet_1.docx]

Supplementary Materials

Normative data for the trail making tests with all participants are available in Appendices 1a, 1b and 1c. Normative data for trail making tests by gender, education and age group among urban community-dwelling older adults without neurological symptoms are available in Appendices 2a, 2b and 2c. The percentiles among all participants are shown in Appendices 3a, 3b, 3c and 3d. The percentiles among urban community-dwelling older adults without neurological symptoms are shown in Appendices 4a, 4b, 4c and 4d. Values for subgroups with sample sizes (n) of less than 50 do not meet the criteria for normative data, but are presented as reference information. Data for ages 90+ in the percentile tables were not split into subgroups due to small sample size.

Appendix 1a. Normative data for Trail Making Test Part A by gender, education, MMSE-J for each age group among urban community-dwelling older adults.

Appendix 1b. Normative data for Trail Making Test Part B by gender, education, MMSE-J for each age group among urban community-dwelling older adults.

Appendix 1c. Normative data for delta TMTs (A-B and B/A) by gender, education, MMSE-J for each age group among urban community-dwelling older adults.

Appendix 2a. Normative data for Trail Making Test Part A by gender, education and age group among urban community-dwelling older adults without neurological symptoms.

Appendix 2b. Normative data for Trail Making Test Part B by gender, education and age group among urban community-dwelling older adults without neurological symptoms.

Appendix 2c. Normative data for delta TMTs (A-B and B/A) by gender, education and age group among urban community-dwelling older adults without neurological symptoms.

Appendix 3a. Percentiles for Trail Making Test Part A by gender, education, for each age group among urban community-dwelling older adults.

Appendix 3b. Percentiles for Trail Making Test Part A by gender, education, for each age group among urban community-dwelling older adults.

Appendix 3c. Percentiles for delta TMT (B-A) by gender, education, for each age group among urban community-dwelling older adults.

Appendix 3d. Percentiles for delta TMT (B/A) by gender, education, for each age group among urban community-dwelling older adults.

Appendix 4a. Percentiles for Trail Making Test Part A by gender, education, for each age group among urban community-dwelling older adults without neurological symptoms.

Appendix 4b. Percentiles for Trail Making Test Part B by gender, education, for each age group among urban community-dwelling older adults without neurological symptoms.

Appendix 4c. Percentiles for delta TMT (B-A) by gender, education, for each age group among urban community-dwelling older adults without neurological symptoms.

Appendix 4d. Percentiles for delta TMT (B/A) by gender, education, for each age group among urban community-dwelling older adults without neurological symptoms.
